# On the Performance of an Aerosol Electrometer with Enhanced Detection Limit

**DOI:** 10.3390/s18113889

**Published:** 2018-11-12

**Authors:** Yixin Yang, Tongzhu Yu, Jiaoshi Zhang, Jian Wang, Wenyu Wang, Huaqiao Gui, Jianguo Liu

**Affiliations:** 1 Key Laboratory of Environmental Optics and Technology, Anhui Institute of Optics and Fine Mechanics, Chinese Academy of Sciences, Hefei 230031, China; yxyang@aiofm.ac.cn (Y.Y.); tzyu@aiofm.ac.cn (T.Y.); wangjiansnbf@163.com (J.W.); wywang@aiofm.ac.cn (W.W.); hqgui@aiofm.ac.cn (H.G.); 2 Science Island Branch of Graduate School, University of Science and Technology of China, Hefei 230026, China; 3 CAS Center for Excellence in Regional Atmospheric Environment, Institute of Urban Environment, Chinese Academy of Sciences, Xiamen 361021, China

**Keywords:** aerosol electrometer, sub-femtoamp noise, detection efficiency, low detection limit

## Abstract

An aerosol electrometer with enhanced detection limit was developed for measuring the collected particles electrical current ranging from −50 pA to 50 pA with no range switching necessary. The detection limit was enhanced by suppressing the electric current measurement noise and improving the detection efficiency. A theoretical model for the aerosol electrometer has been established to investigate the noise effect factors and verified experimentally. The model showed that the noise was a function of ambient temperature, and it was affected by the characteristics of feedback resistor and operational amplifier simultaneously. The Faraday cup structure of the aerosol electrometer was optimized by adopting a newly designed cup-shaped metal filter which increased the surface area of the cup; thus the particle interception efficiency was improved. The aerosol electrometer performance-linearity, noise and the particle detection efficiency, were evaluated experimentally. When compared with TSI-3068B, a 99.4% ($R^{2}$) statistical correlation was achieved. The results also showed that the root mean square noise and the peak-to-peak noise were 0.31 fA and 1.55 fA, respectively. The particle detection efficiency was greater than 99.3% when measuring particle diameter larger than 7.0 nm.

## 1. Introduction

Aerosol electrometers (AE), which mainly consist of an electrometer and a Faraday cup-containing filter, is the simplest form of electrical aerosol measurement instrument used in aerosol studies. They have been widely used in characterizing condensation particle counters (CPC) [1,2,3,4], determining the size distribution with a differential mobility analyzer (DMA) [5,6,7,8], and directly measuring net charge on atmospheric ions or charged aerosols [9,10,11,12]. AEs have also been employed as a primary standard of the particle number concentration, since it depends only on the volume flow of aerosol and electric current induced by charged particles, which are traceable to the primary standard for flow and electric current [13,14,15].

Currently, the challenge of AEs being used in different fields is their detection limit, which is mainly affected by electric current measurement noise and the particle detection efficiency. It becomes the general source of uncertainty at low particle number concentration. For instance, the CPC counting efficiency curve should be calibrated by taking the concentration ratio of the CPC to the AE for particles with different particle sizes. To avoid large coincidence errors in CPC, the particle number concentration cannot be too high. Therefore, AEs with low electric current measurement noise and high particle detection efficiency are in demand for the sake of high signal to noise ratio. In the work of Intra and Tippayawong [10,11], an AE with the output range of 10 fA to 10 pA was designed and evaluated to measure and sample the atmospheric ions and charged aerosols. However, they neglected to test the electric current measurement noise, and the analog voltage output of the electrometer is easily interfered by the ambient noise even when submerged. Additionally, electric current noise, which would affect the detection limit, was also generated by other components in the aerosol electrometer aside from the electrometer itself when temperature variations. Mora et al. designed two fast electrometer circuits and installed them in a Faraday cup [15]. One of the electrometers ($10^{12}$ V/A) exhibited an excellent noise level, which was 0.1 fA at 1 Hz sample frequency. However, the long-term output drift was about 5.7 fA per hour over a 7-h period. The drift would accumulate when taking long-term measurements. Hence, the AE must be zeroed every once in a while. Commercial instruments were also employed in constructing the AE [14,15]. For instance, a Keithley 6430 sub-femtoamp remote source meter (Keithley Instruments Inc., Cleveland, OH, USA) has been widely used as an electrometer in AE, since it can measure electric current from 1pA range (with just 0.4 fA peak to peak noise) up to the 100 mA range. However, it should note that the noise level of these kinds of AEs was normally much higher than the noise level of the Keithley 6430 itself. The reasons are two fold. One is that both the ambient temperature fluctuation and the temperature difference between Faraday cup and Keithley 6430 will cause noise, which is then added to the desired current and caused errors. Second, although the Faraday cup was connected to the Keithley 6430 by a low noise cable, vibration noise can’t be completely eliminated. In our previous work [16], an electrometer was designed for measuring electrical current from high number concentration aerosol particles ranging from −500 pA to +500 pA. The long-term output drift was within 5.2 fA over a 20-h period, and the root mean square (RMS) noise was 1.04 fA, however, the designed AE in that study was focus more on wide dynamic range, and was lack of testing for the particle detection efficiency.

The aim of this study was, therefore, to develop an optimized aerosol electrometer with enhanced detection limit. The purpose of this study was to analyze the effect factors on noise theoretically and experimentally for sake of reducing noise, and to improve the particle detection efficiency by adopting a high efficiency cup-shaped metal filter. At last, the correlation of the designed AE was evaluated by compared with a commercial one, and the performance of the designed AE, such as linearity, noise and the particle detection efficiency, was assessed by a series of experiments.

## 2. Materials and Methods

### 2.1. Design and Modeling

**Figure 1:**
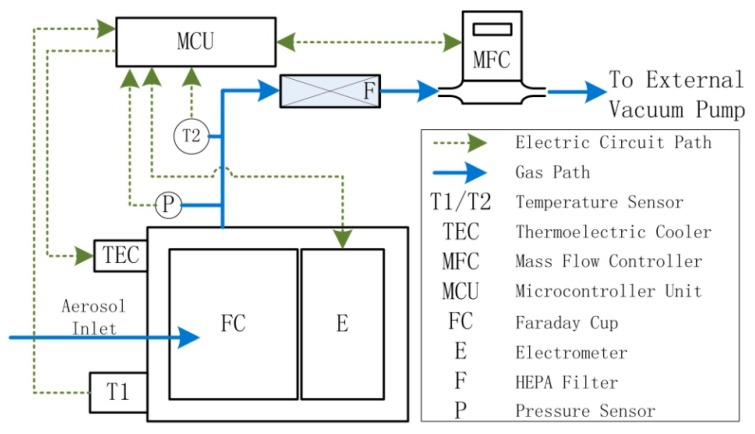
Schematics diagram of the AE.

Figure 1 showed the schematic diagram of the designed AE. It mainly consists of a solid-state electrometer with a faraday cup (FC), following by a HEPA filter and a mass flow controller (Sevenstar CS200, NAURA Technology Group Co., Ltd., Beijing, China). A thermoelectric cooler (TEC) and a temperature sensor (thermistor) have also been used to guarantee the temperature stability of the FC and the electrometer. A micro programmed control unit (MCU) is used to control the whole system. Additionally, temperature and pressure sensors have also been used to convert the mass flow to volume flow.

**Figure 2:**
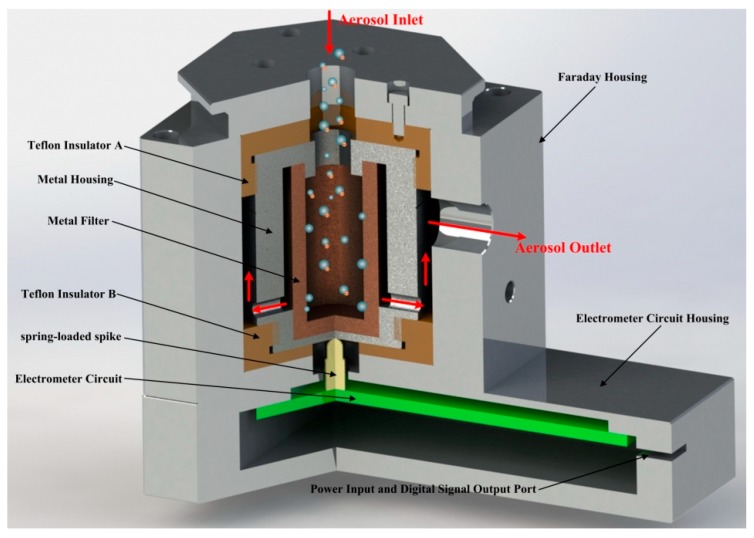
The schematics diagram of the FC and electrometer.

Figure 2 showed the schematic diagram of the FC and electrometer. As shown, charged particles were collected using a high efficiency conductive filter. After that, electric current generated and was measured by a solid-state electrometer. The particle number concentration can then be calculated due to the proportional relationship of the charge collection rate on the filter and the electric current. Therefore, the detection limit of particle number concentration is mainly subject to the particle detection efficiency and the electric current measurement noise. Compared with our previous work [16], the FC and electrometer have been optimized to increase the collection efficiency and decrease the electric current measurement noise. In the FC, the surface area of the filter has been increased by using a cup-shaped metal filter instead of disc-shaped metal filter for sake of improving the collecting efficiency. As stated in work [16], the metal filter is sintered copper powder filter element. In order to increase the collection efficiency by increasing particle interception efficiency, 300-mesh spherical copper powder has been used to produce the metal filter. Moreover, the structure of the FC has modified by threading the cap of the metal housing, the insulator A and the cap of shielding outer case together for easier maintenance.

**Figure 3:**
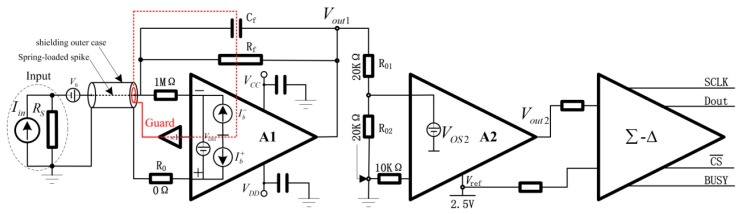
The schematics diagram of electrometer circuit.

An electrometer circuit with sub-femtoamp noise and no range switching necessary has been developed to measure the ultra-low current. Moreover, a theoretical model have been proposed to analyze the effect factors of electric current measurement noise. The electrometer circuit used in the AE, which is similar with our previous work [16], has been shown in Figure 3. It consists of a current-voltage converter, a level converter and a high resolution analog-digital converter (ADC).

In the current-voltage converter, a feedback resistor ($R_{f}$) with high value and low temperature coefficient, a mica capacitor ($C_{f}$) with high insulation resistance and an operational amplifier (A1), which has femtoampere ($10^{-15}$ A) level input bias current ($I_{b}$) was employed. The input ultra-low current ($I_{\mathrm{in}}$) could then be converted to voltage ($V_{\mathrm{out}1}$) after it flowed through the high resistance value feedback resistor ($R_{f}$). T he output voltage ($V_{\mathrm{out}1}$) can therefore be stated as [17]:

(1) $$V_{\mathrm{out}1}=-I_{\mathrm{in}}R_{f}+(V_{\mathrm{os}1}+V_{0})\left(1+\frac{R_{f}}{R_{S}}\right)-I_{b}^{-}R_{f}+I_{b}^{+}R_{0}$$

where $V_{\mathrm{os}1}$ is the input offset voltage of A1; $R_{S}$ is the FC resistance, and typically $R_{f}\ll R_{s}$; $V_{0}$ is the noise voltage of the FC, which caused by thermoelectric voltages among spring-loaded spike, metal housing and metal filter and the triboelectric, piezoelectric, and stored charge effects between the Teflon insulation and the filter holder [18]. The $V_{\mathrm{out}1}$ can then be simplified to:

(2) $$V_{\mathrm{out}1}=-I_{\mathrm{in}}R_{f}+V_{\mathrm{os}1}+V_{0}-I_{b}^{-}R_{f}$$

In the level converter, two precise resistors ($R_{01}$ and $R_{02}$) with the same resistance and low temperature coefficient, and a low noise instrumentation amplifier (A2) have been applied. Moreover, a low-noise voltage reference was used to provide a precision reference voltage (+2.5 V). The converted voltage ($V_{\mathrm{out}1}$), which was ranging from −5 V to +5 V, was convert to range from 0 V to +5 V. Therefore, the output voltage ($V_{\mathrm{out}2}$) after instrumentation amplifier (A2) could be calculated by:

(3) $$V_{\mathrm{out}2}=\frac{R_{02}}{R_{01}+R_{02}}\left(-I_{\mathrm{in}}R_{f}+V_{\mathrm{os}1}+V_{0}-I_{b}^{-}R_{f}\right)+2.5+V_{\mathrm{os}2}$$

where $V_{\mathrm{os}2}$ is the input offset voltage of A2.

In the high resolution analog-digital converter, a 24-bit differential delta-sigma (Δ-Σ) ADC was employed to convert the sensitive analog signal directly to an anti-interference digital signal. As shown, the precision reference voltage (+2.5 V) was also one of the differential input, hence the measurements of the ADC ($V_{\mathrm{out}}$) could be stated as:

(4) $$V_{\mathrm{out}}=R\cdot \left(-I_{\mathrm{in}}R_{f}+V_{\mathrm{os}1}+V_{0}-I_{b}^{-}R_{f}\right)+V_{\mathrm{os}2}$$

where $R=\frac{R_{02}}{R_{01}+R_{02}}$.

Since $R_{01}$ and $R_{02}$ were both precise resistors with the same value and low temperature coefficient, $R=\frac{1}{2}$ and $\frac{\mathrm{dR}}{\mathrm{dT}}=\frac{R_{01}\frac{dR_{02}}{\mathrm{dT}}-R_{02}\frac{dR_{01}}{\mathrm{dT}}}{{\left(R_{01}+R_{02}\right)}^{2}}\to 0$. The converted voltage ($V_{\mathrm{out}}$) could then be simplified to:

(5) $$V_{\mathrm{out}}=-\frac{1}{2}I_{\mathrm{in}}R_{f}+\left(\frac{1}{2}V_{\mathrm{os}1}+\frac{1}{2}V_{0}+V_{\mathrm{os}2}\right)-\frac{1}{2}I_{b}^{-}R_{f}$$

It is commonly accepted that some parameters, including the value of resistors, the input offset voltage of amplifier and the input bias current, are temperature dependent. In order to guarantee the measure accuracy, an electrometer was commonly calibrated in a stabilized temperature. According to the Equation (5), when the temperature of the electrometer was stabilized at $T_{0}$ °C, the measurements of the ultra-low current could be calculated after calibrated:

(6) $$I_{\mathrm{out}}=-\frac{2}{R_{f}\left(T_{0}\right)}\cdot V_{\mathrm{out}}+\frac{V_{\mathrm{os}1}\left(T_{0}\right)+V_{0}\left(T_{0}\right)+2V_{\mathrm{os}2}\left(T_{0}\right)}{R_{f}\left(T_{0}\right)}-I_{b}^{-}\left(T_{0}\right)$$

where $I_{\mathrm{out}}$ and $V_{\mathrm{out}}$ are the measurements of the ultra-low current after calibrated and the measurements of ADC, respectively; $V_{\mathrm{os}1}\left(T_{0}\right)$ and $V_{\mathrm{os}2}\left(T_{0}\right)$ are the input offset voltage of A1 and A2 at $T_{0}$ °C, respectively; $I_{b}^{-}\left(T_{0}\right)$ is the input bias current of A1 at $T_{0}$ °C, and $R_{f}\left(T_{0}\right)$ is the resistance value of the feedback resistor at $T_{0}$ °C.

Combined with the Equations (5) and (6), the measurements of the ultra-low current could be calculated when the operating temperature is T °C, and it could be expressed as:

(7) $$I_{\mathrm{out}}=K_{f}\cdot I_{\mathrm{in}}+\frac{\left[V_{\mathrm{os}1}\left(T_{0}\right)-V_{\mathrm{os}1}\left(T\right)]+[V_{0}\left(T_{0}\right)-V_{0}\left(T\right)]+2[V_{\mathrm{os}2}\left(T_{0}\right)-V_{\mathrm{os}2}\left(T\right)\right]}{R_{f}\left(T_{0}\right)}-\left[I_{b}^{-}\left(T_{0}\right)-K_{f}\cdot I_{b}^{-}\left(T\right)\right]$$

where the symbol $K_{f}$ used in the equation is defined as $K_{f}=\frac{R_{f}\left(T\right)}{R_{f}\left(T_{0}\right)}$; $I_{b}\left(T\right)$ is the input bias current of A1 at T °C, and $R_{f}\left(T\right)$ is the resistance value of the feedback resistor at T °C; $V_{\mathrm{os}1}\left(T\right)$ and $V_{\mathrm{os}2}\left(T\right)$ are the input offset voltage of A1 and A2 at T °C, respectively.

Suppose that the temperature coefficient itself does not vary too much with temperature, the resistance value $R_{f}\left(T\right)$ at T °C could be estimated by a linear approximation [19]:

(8) $$R_{f}\left(T\right)=R_{f}\left(T_{0}\right)\left[1+\alpha \left(T-T_{0}\right)\right]$$

where α is the temperature coefficient of the $R_{f}$.

Therefore, $K_{f}=\frac{R_{f}\left(T\right)}{R_{f}\left(T_{0}\right)}=\frac{R_{f}\left(T_{0}\right)\left[1+\alpha \left(T-T_{0}\right)\right]}{R_{f}\left(T_{0}\right)}=1+\alpha \left(T-T_{0}\right)$, and the Equation (7) can then be expressed as

(9) $$I_{\mathrm{out}}=\left[1+\alpha \left(T-T_{0}\right)\right]\cdot I_{\mathrm{in}}+B\left(T\right)$$

where $B\left(T\right)=\frac{\left[V_{\mathrm{os}1}\left(T_{0}\right)-V_{\mathrm{os}1}\left(T\right)]+[V_{0}\left(T_{0}\right)-V_{0}\left(T\right)]+2[V_{\mathrm{os}2}\left(T_{0}\right)-V_{\mathrm{os}2}\left(T\right)\right]}{R_{f}\left(T_{0}\right)}-\left\{I_{b}^{-}\left(T_{0}\right)-\left[1+\alpha \left(T-T_{0}\right)\right]\cdot I_{b}^{-}\left(T\right)\right\}$. 

Zero offset, which is a gradual change of output result with no input signal, can be identified as B(T). According to the Equation (9), the offset is normally specified as a function of temperature, and is affected by the characteristics of feedback resistor and operational amplifier. It will causes an error by adding to the input signal even it had been zero checked, when temperature fluctuate. In order to decrease the electric current measurement noise, zero offset should be controlled. Apart from the strategies mentioned in [16], zero offset can also be decreased by increase the value of feedback resistor. However, it will decrease the dynamic range, which is defined as a decibel logarithmic value of the ratio of the largest and smallest signal values. Therefore, a tradeoff between the detecting limit and the dynamic range should be considered adequately under different practical applications. Additionally, the limit noise reference—Johnson noise is also specified as a function of temperature and is also affected by the characteristics of feedback resistor [19]. In this design, a metal oxide ultra-high resistor (model RX-1M1009FE, 100 GΩ, OHMITE, Warrenville, IL, USA) with low tolerance (±1%) and low temperature coefficient (±50 ppm/°C) has been used in the circuit to improve the detecting limit.

It is commonly accepted that the current measured by the electrometer has a proportional relationship to the input aerosol number concentration N ($\mathrm{particle}/{\mathrm{cm}}^{3}$), and can be calculated as [13]:

(10) $$I_{\mathrm{out}}=N\cdot n_{p}\cdot e\cdot \eta_{\mathrm{FC}}\cdot Q$$

where $n_{p}$ is the average number of e per particle; e is the elementary unit of charge; $\eta_{\mathrm{FC}}$ is the particle detection efficiency; and Q (${\mathrm{cm}}^{3}/s$) is the volumetric flow rate of aerosol.

Combined with the Equations (6) and (10), the input aerosol number concentration N ($\mathrm{particle}/{\mathrm{cm}}^{3}$), can be calculated as:

(11) $$N=\frac{-\frac{2}{R_{f}\left(T_{0}\right)}\cdot V_{\mathrm{out}}+\frac{V_{\mathrm{os}1}\left(T_{0}\right)+V_{0}\left(T_{0}\right)+2V_{\mathrm{os}2}\left(T_{0}\right)}{R_{f}\left(T_{0}\right)}-I_{b}^{-}\left(T_{0}\right)}{n_{p}\cdot e\cdot \eta_{\mathrm{FC}}\cdot Q}$$

Therefore, the electrometer and the volumetric aerosol flow rate should be calibrated firstly. As our previous work [16], the keithley 6221 current source (Keithley Instruments) has been introduced in the calibration of the electrometer. The volumetric aerosol flow rate has also been calibrated by a NIST-traceable electronic bubble flowmeter (Gilian^®^ Gilibrator™2, Sensidyne Inc., Clearwater, FL, USA). Additionally, the particle detection efficiency ($\eta_{\mathrm{FC}}$) was evaluated below.

### 2.2. Experiment Setup for Evaluating the AE

**Figure 4:**
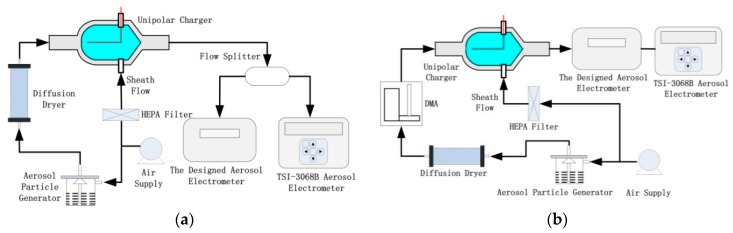
Schematics diagram of the experiment setup for evaluating the AE. (**a**) The experiment setup for comparing the designed AE and the TSI-3068B; (**b**) the experiment setup for evaluating the particle detection efficiency of the designed AE.

Figure 4 showed the schematic diagram of the experiment setup for evaluating the AE. Following this setup, a comparison between the AE and a commercial aerosol electrometer (TSI-3068B) can be achieved in Figure 4a, and then the particle detection efficiency ($\eta_{\mathrm{FC}}$) has been evaluated in Figure 4b. To be mentioned, the TSI-3068B has the measurement range of ±12.5 pA, ±2% of reading or ±5 fA current accuracy (whichever is greater), and <1 fA RMS noise at 1s average. The drift is ±2 fA at an average of 1s over 24 h at environment conditions of 5 °C and 50% RH and 35 °C and 50% RH, or ±2 fA at an average of 1 s over 1 h at environment conditions of 35 °C and 90% RH. The maximum data rate is 1 Hz, and the particle size range is 2 to 5000 nm. Normally, the filter in the FC is adjudged an absolute filter that collects the charged particles from the sample flow. According to Gauss’ law, the charge collected on the FC is the induced charge, which means that all the charged particles entered the FC can be detected with 100% detection efficiency. However, the absolute filter does not really exist which means that the detection efficiency cannot all be 100% for different size particles. Generally, the collection efficiency of the FC is defined as the ratio of particle number collected on the FC to the particle number entered the FC. However, the particle detection efficiency is not equivalent to the collection efficiency, since charged particles escaped from the FC would also contact with the metal filter in a certain probability and lose charges and then be detected. Thus, a more rigorous definition of the particle detection efficiency ($\eta_{\mathrm{FC}}$) is the ratio between electric current caused by charged particles collected on the FC and electric current caused by charged particles entered the FC.

The setup mainly consists of an air supply module, an aerosol particle generator, an unipolar charger, a commercial aerosol electrometer (TSI-3068B), and the designed AE. In the air supply module, an air compressor has been used, following by a diffusion dryer, a high efficiency particulate-free air (HEPA) filter and an electrical precipitator in turn. The clean dry air flow is supplied for aerosol particle generator and unipolar charger. A MetOne 255 atomizer (Met One Instruments Inc., Los Angeles County, CA, USA) has been employed in the aerosol particle generator to generate sodium chloride (NaCl) particles for comparison between the AE and the TSI-3068B. For evaluation of the particle detection efficiency, a MetOne 255 atomizer has been employed to generate sodium chloride (NaCl) particles or spherical polystyrene latex (PSL) microspheres (Duke Scientific Inc., Palo Alto, CA, USA), and then a electrostatic classifier (TSI-3082, TSI Inc., Shoreview, MN, USA) equipped with a 1-nm TSI-3086 Differential Mobility Analyzer (DMA) has been used to generate sub-micrometer monodisperse particles. These particles were then delivered to a diffusion dryer for water removing, and to the unipolar charger in turn. The unipolar charger was designed and evaluated previously [20]. As showed in Figure 4a, the designed AE and the TSI-3068B were compared with each other. A flow splitter (TSI-3708) was used to direct the charged particles to the designed AE and the TSI-3068B (other outlets were closed) simultaneously and evenly. The flow rate of TSI-3068B and the designed AE were both set to be 2 lpm. As showed in Figure 4b, the particle detection efficiency of the designed AE was evaluated by putting the designed AE and the TSI-3068B in series. The electric current ($I_{\mathrm{FC}}$), which caused by charged particles collected on the FC, was measured. The electric current ($I_{\mathrm{lost}}$), which caused by charged particles escaped from the FC, was measured by TSI-3068B simultaneously. In this scheme, the flow rate was set to be 2.5 lpm. The particle detection efficiency could then be calculated as $1-\frac{I_{\mathrm{lost}}}{I_{\mathrm{FC}}}$. To keep the same particle diffusion losses in Figure 4a, the flow paths were symmetrical. It means that the flow rates and conductive silicone tube lengths from the flow splitter to the designed AE/TSI-3068B inlet are the same. In the Figure 4b, the conductive silicone tube with 1/4 inch inner diameter and 5 cm length was used between the output of the designed AE and the inlet of the TSI-3068B for sake of alleviating particle losses. Additionally, the particle number concentration should be as high as possible for sake of achieving high signal-to-noise ratio of the designed AE and TSI-3068B.

## 3. Results

### 3.1. Results of Calibration

The current measurement of the AE was calibrated by the Keithley 6221 ranging from −50 pA to +50 pA with 1 s sampling time. It should be mentioned that the temperature of the electrometer was stabilized at 27 °C. All sourced current was hold 60 s followed by 20 measurements at each calibration points.

**Figure 5:**
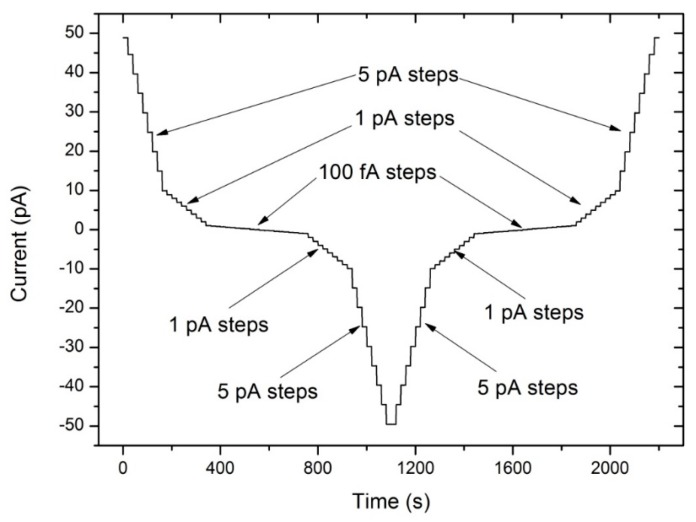
The calibration scheme of AE at range of −50 pA to +50 pA.

As shown in Figure 5, the measurement sequence started with currents in the +pA range, followed by +fA range, the zero, −fA range, −pA range in turn, and finally return to +pA range as a closing set. Currents in the pA range were from ±10 pA to ±50 pA at 5 pA steps, and 1pA steps at the range of ±1pA to ±10 pA. Current in the fA range were from 0 fA to ±1 pA at 100 fA steps. 

**Figure 6:**
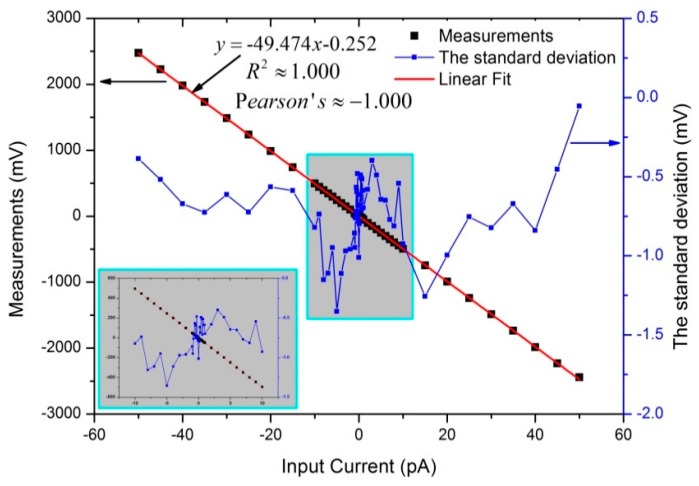
Results of linearity calibration.

The calibration results and its linear fit are shown in Figure 6. Additionally, the standard deviation of each calibration points are also calculated. The detailed results ranging from −10 pA to +10 pA have been enlarged and shown in Figure 6. The results confirmed the theoretical equation described in Equation (5). The excellent fit and good linear relationship were further confirmed by the Adj. R-Square (≈1.000) and the Pearson’s correlation coefficient (≈−1.000). Compared to Equation (5), the actual value of ultra-high feedback resistor $R_{f}$ was 98.948 GΩ. According to Equation (6), the designed AE can be calibrated as $I_{\mathrm{out}}\left(\mathrm{pA}\right)=-0.020\cdot V_{\mathrm{out}}\left(\mathrm{mV}\right)-0.005$, and the algorithm was loaded in MCU.

### 3.2. Noise of the Aerosol Electrometer

**Figure 7:**
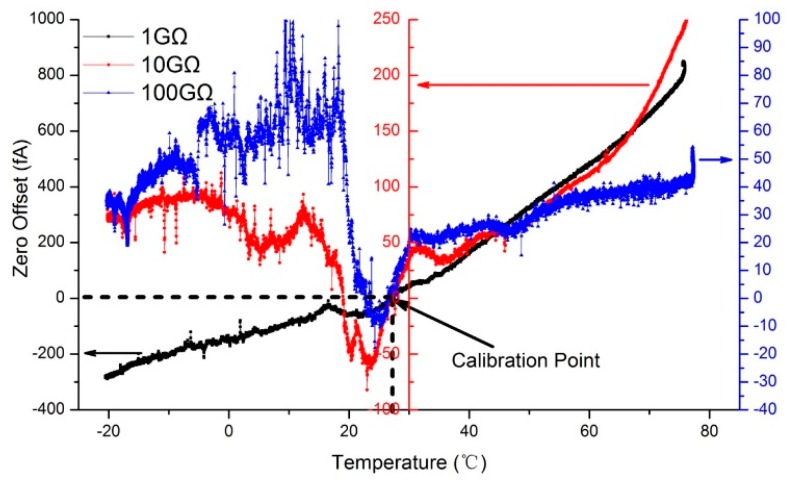
Typical zero offset due to temperature variations of 1 GΩ, 10 GΩ and 100 GΩ.

With the foregoing discussion, the electric current measurement noise is subject to zero offset and Johnson noise. According to the model for the designed AE, both the zero offset and the Johnson noise are specified as a function of ambient temperature and affected by the characteristics of feedback resistor. In this study, an experiment was carried out to test the temperature effect on the zero offset of the designed AE. Three electrometers with different dynamic range have been used. It means that three different feedback resistances were adopted, which were 1 GΩ (TE Connectivity model HB11G0FZRE), 10 GΩ (OHMITE model HVC4020V1008KET) and 100 GΩ (OHMITE model RX-1M1009FE). In the experiment, the designed AE was operated from −25 °C to 80 °C with a 2 °C/min heating rate in a commercial high-low temperature test chamber (HKT705P-10, Hongze, Shanghai, China). The temperature in the test chamber was measured by a platinum resistance temperature sensor (PT100). A SHT11temperature sensor (Sensirion, Staefa ZH, Switzerland) was installed inside the electrometer and recorded the temperature variation from −20 °C to 75 °C. The SHT11 is Sensirion’s family of surface mountable relative humidity and temperature sensors with the typical accuracy of ±0.4 °C. The designed AE was also calibrated at 27 °C. Corresponding to the actual temperature of the AE, zero offset was acquired when there was no charged aerosol input. Typical zero offset due to temperature variations of three different electrometers are shown in Figure 7.

**Figure 8:**
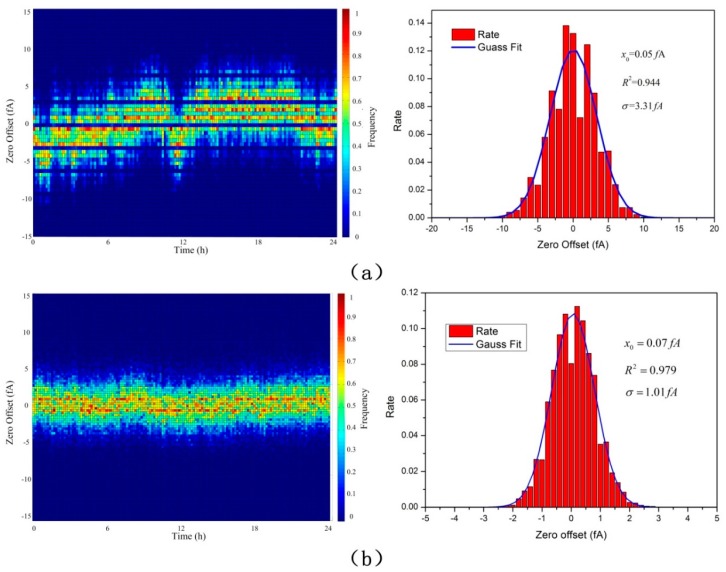

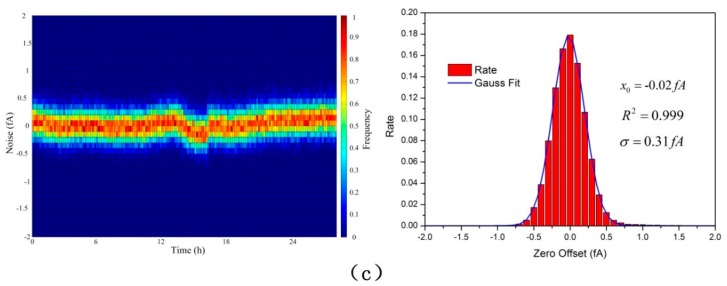
The Noise of the three AEs after achieving thermal stability. (**a**) The AE with 1 GΩ feedback resistance; (**b**) the AE with 10 GΩ feedback resistance; (**c**) the AE with 100 GΩ feedback resistance.

As shown, the x-axis represents the actual temperature of the AE and the three y-axis represent the zero offset of three AEs with different feedback resistance (1, 10 and 100 GΩ, respectively). Corresponding to the entire temperature changing process from −20 °C to 75 °C, zero offset change process from −300 fA to 900 fA, from −80 fA to 250 fA, and from −20 fA to 100 fA, corresponding to the 1 GΩ, 10 GΩ and 100 GΩ feedback resistance. The results showed good agreement with the theoretical discussion as before. To minimized errors due to zero offset, it is essential to operate the FC and electrometer in a thermally stable environment. In the designed AE, a TEC and a temperature sensor have also been used. Combined with proportion integration differentiation (PID) control algorithm that loaded in the MCU, the FC and electrometer can be controlled in a proper temperature. In view of high humidity aerosol condensation caused by a sharp fall of temperature, the temperature of the FC and electrometer were controlled to be 5 °C higher than ambient temperature and then zeroed after achieving thermal stability. After that, clear air was supplied to the AEs, and the noise of the three AEs with 1, 10 and 100 GΩ feedback resistance have been tested more than 24 h in 27 °C. The results are shown in Figure 8.

As shown, the noise of the three AEs after achieving thermal stability obeys Gaussian distribution. The RMS noise with respect to the mean were 3.31 fA, 1.01 fA and 0.31 fA respectively. They were very close to the theoretical limit of the electric current noise–Johnson noise. Typically, the peak-to-peak noise is within five times of the RMS noise more than 99% of the time [18]. It has been, therefore, calculated to be 16.55 fA, 5.05 fA and 1.55 fA, respectively. The dynamic range can then be calculated to be $20\log_{10}\frac{5\;\mathrm{nA}\times 2}{16.55\;\mathrm{fA}}\approx 115.6\;\mathrm{dB}$, $20\log_{10}\frac{500\;\mathrm{pA}\times 2}{5.05\;\mathrm{fA}}\approx 105.9\;\mathrm{dB}$ and $20\log_{10}\frac{50\;\mathrm{pA}\times 2}{1.55\;\mathrm{fA}}\approx 96.2\;\mathrm{dB}$, respectively.

### 3.3. Particle Detection Efficiency of the AE

**Figure 9:**
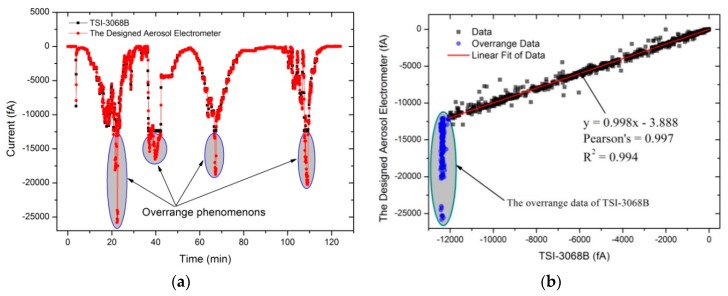
Results of the designed AE compared to the TSI-3068. (**a**) The measurement results; (**b**) the correlation.

Following the experimental setup shown in Figure 4b, an experiment was carried out to test the particle detection efficiency of the AE. Firstly, the performance of the AE was evaluated by comparing with a commercial aerosol electrometer (TSI-3068B) according to the experimental setup showed in Figure 4a. The charged particle number concentration was regulated by adjusting sheath flow, which was embed in MetOne 255 (Met One Instruments Inc., Los Angeles County, CA, USA). Figure 9a showed the measurement results for the set of 125 min. From the data in Figure 9b, a 99.4% (*R*^2^) statistical correlation was obtained between the TSI-3068B and the designed AE by sampling charged particles simultaneously. Additionally, the overrange data shown in Figure 9b could be attributed to the limited dynamic range of the TSI-3068B.

**Figure 10:**
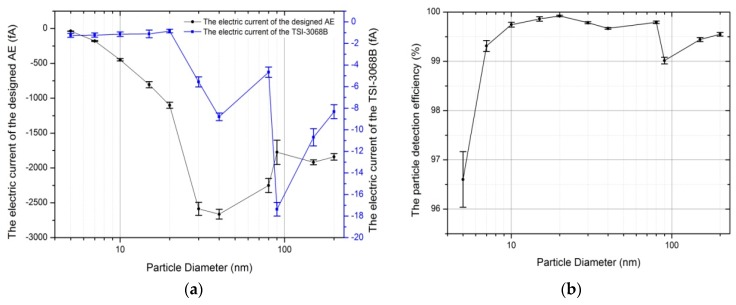
Results of evaluating the particle detection efficiency. (**a**) The electric current of the designed AE and the TSI-3068B; (**b**) the results of the particle detection efficiency.

After that, the particle detection efficiency was tested by connecting the designed AE and the TSI-3068B in series. PSL microspheres were used to generate particles with diameter larger than or equal to 30 nm by a MetOne 255 atomizer (Met One Instruments Inc., Los Angeles County, CA, USA). NaCl particles were used to generate particles with diameter smaller than 30 nm by a MetOne 255 atomizer combined with TSI-3082 and TSI-3086. The measurement results showed in Figure 10. As shown in Figure 10b, the particle detection efficiency was more than 99.3% when particle diameter larger than 7.0 nm. It was decreased to 96.6% when particle diameter was 5.0 nm. Note that the electric current measured by the designed AE and the TSI-3068B were less than 100 fA and 2 fA, respectively, at 5.0 nm in the Figure 10a. It mean that the number concentration of particles produced was not high enough to ensure the accuracy measurement of the particle detection efficiency, and the reading of the TSI-3068B (2 fA) was about to reach the signal to noise limit of the TSI-3068B. Therefore, the particle detection efficiency may be contained considerable errors at 5.0 nm.

## 4. Conclusions

An aerosol electrometer with enhanced detection limit has been developed and characterized at the range of −50 pA to 50 pA with no range switching necessary. In this study, the detection limit was enhanced by suppressing the electric current measurement noise and improving the detection efficiency. Firstly, a theoretical model of the designed AE was established to investigate the noise effect factors and verified experimentally. It showed that the electric current measurement noise was a function of temperature, and affected by the characteristics of feedback resistor and operational amplifier simultaneously. Therefore, apart from the strategies in [16], a 100 GΩ metal oxide ultra-high resistor has been used to suppress the electric current measurement noise. Secondly, the particle detection efficiency has been improved by optimizing the structure of the FC. The cup-shaped metal filter, which produced by 300-mesh spherical copper powder, has been used to improve the collecting efficiency by increasing the surface area of the filter and particle interception efficiency. Finally, the AE performance-linearity, noise and the particle detection efficiency were evaluated experimentally. The RMS noise and the peak-to-peak noise were 0.31 fA and 1.55 fA, respectively, which were significantly improved when compared to our previous work [16]; meanwhile, the detection efficiency was more than 99.3% when the particle diameter larger than 7.0 nm. It should be mentioned that large errors may be caused when measuring total number concentration due to a lack of detection efficiency for particles with diameters smaller than 7.0 nm. Therefore, we will further improve and test this aspect in the future. Furthermore, quantitative measurement of particle number concentration will be carried out in combination with a unipolar charger, which has been designed and evaluated previously [20].
